# Successful treatment of fulminant Lyme myocarditis with mechanical circulatory support in a young male adult: a case report

**DOI:** 10.3325/cmj.2017.58.185

**Published:** 2017-04

**Authors:** Željko Župan, Dino Mijatović, Igor Medved, Snježana Kraljić, Jurica Juranić, Berislav Barbalić, Marin Oštrić

**Affiliations:** 1 Department of Anesthesiology, Reanimatology and Intensive Care Medicine, Faculty of Medicine, University of Rijeka, Rijeka, Croatia; 2 Clinic of Anesthesiology and Intensive Care Medicine, University Hospital Center Rijeka, Rijeka, Croatia; 3 Department of Surgery, Faculty of Medicine, University of Rijeka, Rijeka, Croatia; 4 Department of Surgery, Division of Cardiac Surgery, University Hospital Center Rijeka, Rijeka, Croatia

## Abstract

We describe the case of fulminant myocarditis due to Lyme disease and use of mechanical circulatory support (MCS) for the treatment of the Lyme carditis associated with refractory cardiogenic shock. Fulminant Lyme myocarditis in young adult male patient led to a sudden onset of acute, severe biventricular heart failure with progressive cardiogenic shock, and multiorgan failure immediately after admission.

The previously healthy 28-year-old man was admitted to hospital with dyspnea, atrial flutter with 160/min ventricles rate, normotension, cardiomegaly, and incipient cardiogenic pulmonary edema on chest x-ray. Within the next 24 hours, the acute heart failure (AHF) progressed to the refractory cardiogenic shock with severe systemic hypotension, respiratory distress, anuria, liver congestion, and laboratory evidence of extremely high level of the anaerobic metabolism in the arterial blood (pH 7.16; HCO_3_ 12.3 mmol/L; BE -14.6; lactates level 17 mmol/L). The transesophageal echo imaging showed severe dilatation and global biventricular akinesis, with left ventricular ejection fraction of 5%. The diagnosis of acute fulminant myocarditis of unknown etiology was reached. Since the patient did not respond rapidly to vasoactive and supportive therapy, MCS was immediately inserted. Broad differential diagnosis of fulminant myocarditis was considered and disseminated Borrelia infection was serologically confirmed and appropriate antimicrobial therapy was started from the fifth day after admission. MCS used over the next 26 days was successfully integrated with pharmacologic support and artificial ventilation in therapy. The patient was discharged from hospital after 65 days with a complete restoration of bilateral heart ejection fraction.

This case shows that the clinical course of the Lyme carditis can present uncommonly with profound cardiovascular collapse and the MSC implementation should be considered in the early stage of drug resistant hemodynamic instability. Rapid transfer to the cardiac center where the MCS is available for all patients with signs and symptoms of AHF due to confirmed or suspected Lyme carditis would be recommended, as this treatment could be the only life-saving method.

Lyme carditis is a known but very rare complication of confirmed Lyme disease, multisystemic illness caused by *Borelia burgdorferi*. In this tick-borne infection, pathogenic spirochetes are transmitted by certain species of Ixodes ticks, most often in summer and autumn (1). Lyme carditis is usually associated with acute early phase of disseminated Lyme disease when spirochetes invade the tissue of the heart, producing inflammatory and immunomodulated myocarditis (2,3). The most common manifestations of Lyme carditis are atrioventricular block, which can rapidly progress to second- or third-degree heart block, and different supraventricular and ventricular arrhythmias (4). Acute myocarditis associated with acute heart failure (AHF) is a very uncommon manifestation of Lyme carditis, occurring in approximately less than 0.5% of all infected patients (5). It can coexist with other organ manifestations of Lyme borreliosis. Many patients can be asymptomatic or present with mild cardiac symptoms despite hematogenous spread of the Borrelia species and disseminated infection. Although serious cardiovascular complications may occur, Lyme carditis generally has a good prognosis and is usually transient when timely and appropriately treated with antimicrobial therapy (6). Patients with the second- or third-degree of atrioventricular blocks require comprehensive evaluation of cardiac function and continuous monitoring of electrocardiogram. Rarely, patients with advanced heart blocks or life-threatening cardiac arrhythmias require invasive therapeutic procedures, such as temporary pacing or implantable cardioverter-defibrillator (7-9). Adult patients with developing AHF due to systemic Borrelia infection have been treated conservatively with standard evidence based acute heart failure medications (10).

Fulminant Lyme myocarditis characterized with a rapidly progressive clinical course leading to severe AHF and refractory cardiogenic shock has not yet been described. Also, this is the first report on the use of mechanical circulatory support (MCS) during the treatment of Lyme carditis due to profound circulatory collapse and impaired oxygenation despite the use of aggressive pharmacologic therapy and artificial ventilation.

## Case report

A previously healthy 28-year-old man was admitted to the University Hospital Center Rijeka with the symptoms of nausea and general weakness, which had started a week prior to admission and progressed ever since. The initial clinical examination revealed orthopnea and tachypnea, atrial flutter with the ventricular rate of 160/min, blood pressure of 160/90 mm Hg, and cardiomegaly with incipient cardiogenic pulmonary edema on chest x-ray. His past medical and family history in the context of cardiac diseases was non-significant.

Within the next 24 hours, the patient’s clinical course deteriorated to the state of cardiogenic shock with manifest multiple organ failure (MOF). At that point he presented with severe respiratory failure, blood pressure of 50/30 mm Hg, ventricular tachycardia with the rate of 160/min, low cardiac output state with high anaerobic metabolism indices (pH 7.16; HCO_3_ 12.3 mmol/L; base excess (BE) -14.6; arterial blood lactate level 17 mmol/L), bilateral pleural effusions, pulmonary edema, acute renal failure (anuria accompanied by blood urea level of 11.3 mmol/L and serum creatinine of 126 μmol/L), liver congestion, and acute liver failure (aspartate transaminase 4549 IU/mL; alanine transaminase 2033 IU/mL; serum albumin level 24.2 g/L; prothrombin time 0.14 s; activated partial thromboplastin time >999 s; total serum bilirubin 55 μmol/L). The serum levels of troponin T and C-reactive protein were 80 ng/mL and 49 mg/L, respectively, and leukocyte count was 9.9x10^9^/L.

**Figure 1:**
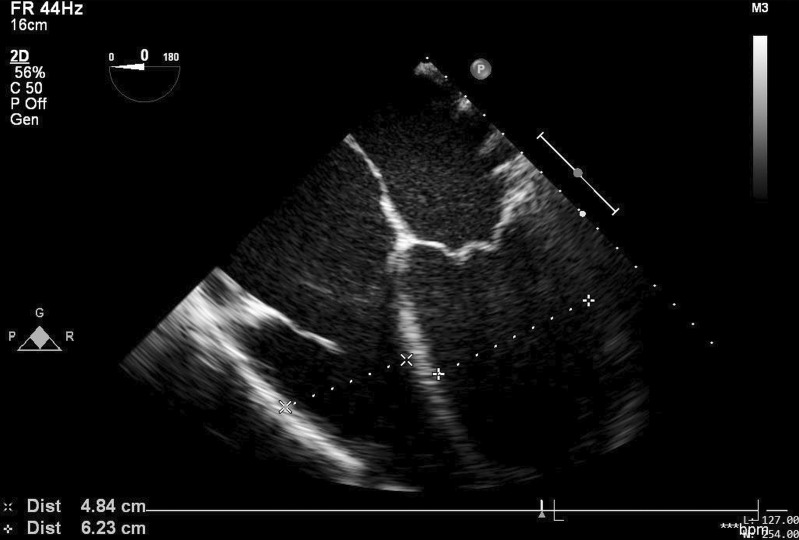
Initial transesophageal echo imaging demonstrating severe dilation of the right ventricle (diameter 4.84 cm) and left ventricle (diameter 6.23 cm) measured in four chambers view.

**Figure 2:**
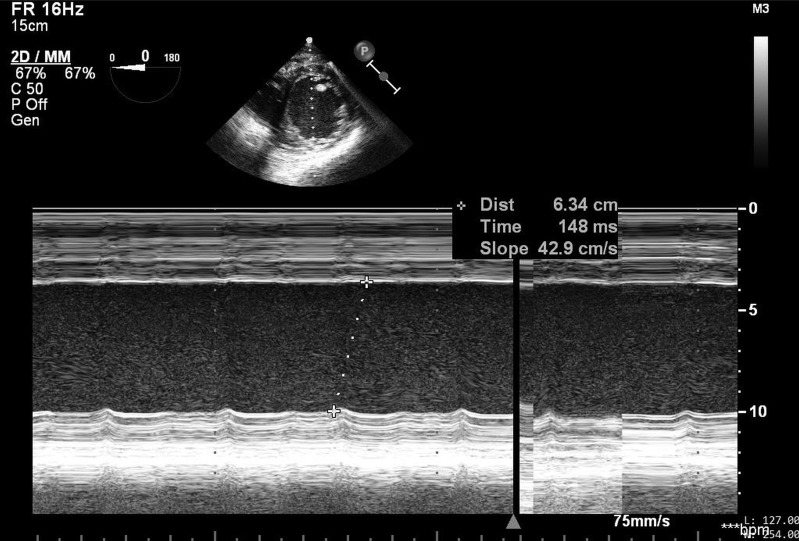
Estimated left ventricular (LV) systolic function (ejection fraction of approximately 5%) according to eye-ball estimation of minimal LV walls excursions in M-mode of transgastric mid-papillary view.

**Figure 3:**
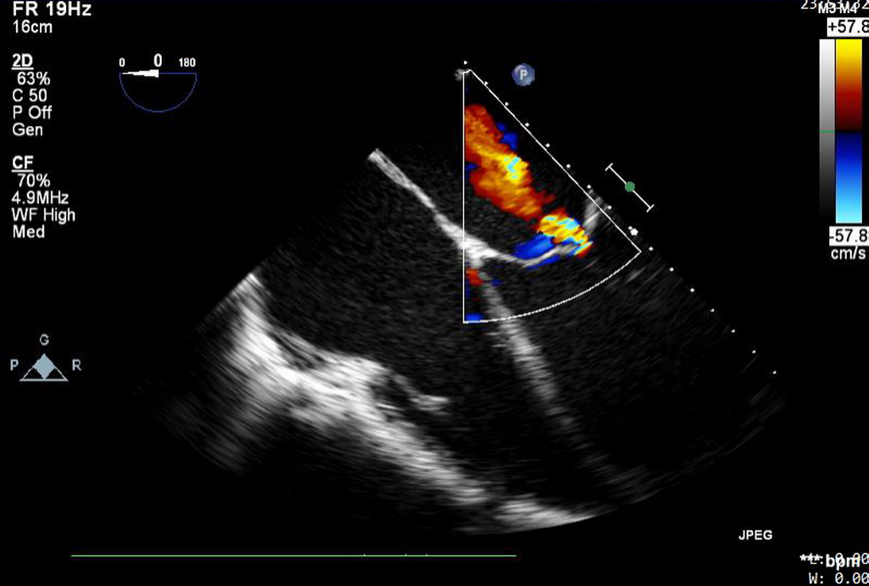
Severe mitral regurgitation on the second day after admission.

**Figure 4:**
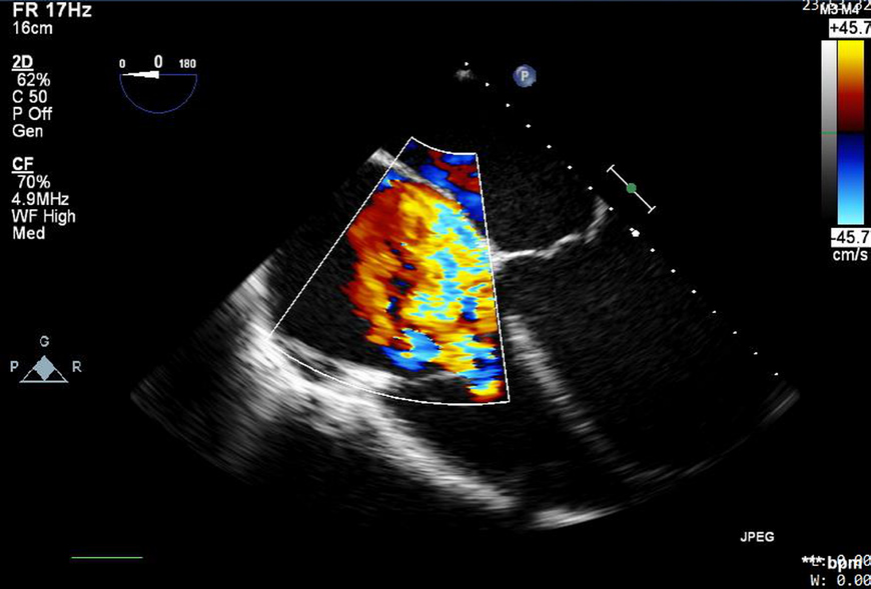
Severe tricuspid regurgitation on the second day after admission.

The patient was transferred to general Intensive Care Unit (ICU), where mechanical ventilation (MV) and multiple vasoactive drug support (norepinephrine 0.4 µg/kg/min i.v., dobutamine 8 µg/kg/min, adrenaline 0.08 µg/kg/min, enoximone 10 µg/kg/min) was immediately started, as well as antiarrhythmic drug amiodarone and diuretic furosemide. Despite these measures, sufficient oxygenation was not achieved as Pao_2_/FiO_2_ ratio was persistently <80 mm Hg and hemodynamic stability was not established. The first transesophageal echo imaging showed a severe dilation (Figure 1) and global akinesis of the left ventricle with ejection fraction (EF) of 5% (Figure 2). There was a concomitant severe mitral regurgitation (Figure 3). The right ventricle was also markedly dilated, showing severe systolic dysfunction and massive tricuspid regurgitation (Figure 4). No other significant pathology was detected, apart from small pericardial effusion.

Coronary angiogram and multislice computed tomography (MSCT) aortography revealed no significant pathology and, taking into account the initial clinical course of the disease and the patient’s history data, the diagnosis of acute dilated cardiomyopathy, possibly caused by fulminant myocarditis of unknown origin, was considered.

A joint decision was made to commence an emergent MCS. Rotaflow centrifugal pump (Maquet) was used for central veno-arterious extracorporeal membrane oxygenation (VA ECMO) upon surgeon’s successful cannulation of the ascendent aorta and right atrium within 24 hours after admission. Additionally, continuous renal replacement therapy (CRRT) was started, incorporated in the ECMO circuit.

The results of the cytological assessment of intraoperatively obtained pericardial fluid were indicative of acute pericardial inflammation with predominant lymphocyte infiltration. Therefore, we empirically included antibiotics piperacillin/tazobactam and antiviremic medication aciclovir into therapy. Hydrocortisone was also introduced from the second day of hospitalization due to patient’s prolonged hemodynamic instability. Due to severe intrinsic coagulopathy, endomyocardial biopsy was not performed.

On the fourth day of treatment, we confirmed the diagnosis of Lyme carditis on the basis of positive serological evaluation of the *Borrelia burgdorferi sensu lato* infection with positive IgM antibodies and subsequent positive Western blot test result. Antimicrobial treatment was revised to parenteral ceftriaxone.

Maximal ECMO-derived blood flow, calculated as body surface area (BSA) × 2.4 L/min, was maintained during the first week of MCS. Optimal systemic perfusion and oxygenation were achieved the day after the ECMO introduction, leading to obvious improvement of organ function. The CRRT was stopped on the eighth day of treatment as the renal function recovered.

On the 10th day of treatment, ECMO flows became progressively impaired. On echocardiography and MSCT, a large mediastinal thrombi were found, compressing the right atrium and right ventricle and causing signs of cardiac tamponade. The patient was referred to an urgent surgical exploration, during which large blood clots were removed. As there was no evidence of significant recovery of the heart function, we decided to insert long term paracorporeal MCS. The pulmonary artery and the left atrium were additionally cannulated and Rotaflow centrifugal pump (Maquet) was applied for right ventricular assisting device (RVAD). Previously placed cannulas in the right atrium and the ascending aorta were checked and kept and CentriMag Blood Pumping System (Levitronix, Thoratec) was used as left ventricular assisting device (LVAD). Maximal flows, initially used, were calculated according to formula BSA × 2.4 L/min for RVAD and 0.8 × BSA × 2.4 L/min for LVAD. A tissue sample of the heart intraoperatively obtained was sent to pathohistological analysis, which confirmed the diagnosis of acute inflammation of the myocard tissue. Hemodynamic stability was achieved with no use of the vasoactive support and another trial of continuous levosimendan (continuous i.v. infusion of 0.1 µg/kg/min, without bolus) was introduced for the second time. A small dose of ACE inhibitor, cardioselective beta-adrenergic blocking agent, and aldosterone were administered enterally via nasogastric tube for additional pharmacological treatment of AHF.

During the next two weeks, the LVAD and RVAD support was maintained. There were signs of gradual biventricular heart recovery. Echocardiogram revealed full recovery of the right heart function with no tricuspid regurgitation. All walls of the left ventricule started to contract up to a level of EF of 20%-30%. The levels of N-terminal pro b-type natriuretic peptide (NT-proBNP) decreased from 1151 to 440 pg/mL. The flows on RVAD and LVAD were reduced gradually to 70% of maximal support. By the end of the third week of critical care treatment, the parameters obtained from Pulse index Continuous Cardiac Output (PiCCO) monitoring revealed further improvement of heart function and circulation, with further intentional reduction of RVAD and LVAD flows. Regarding evidences of satisfactory recovery of the heart, on the 24th day the patient was weaned off RVAD and decannulated. The left ventricle function recovered further to EF of 45%, with no apparent mitral regurgitation evidence at the echocardiogram. The patient was weaned off LVAD and decannulated two days later. In total, MCS was maintained for the first 26 days of critical care management.

The patient was successfully weaned off MV and extubated on the 32nd day of treatment with no signs of any neurological damage. The patient was kept in the ICU for the next 18 days. During that period, he was gradually subjected to active physical rehabilitation and full oral nutrition. He was immobile for 36 days and lost 25 kg of his body weight during that time. The muscle atrophy and mild to moderate degree of large joints contractures were apparent. Relatively small pressure ulcers were present only in the occipital and sacral regions. The last echocardiogram revealed complete recovery of heart morphology and function. The patient was discharged from the hospital after 65 days of treatment.

The clinical course of the patient was complicated by two episodes of severe bleeding from chest with the development of disseminated intravascular coagulopathy that required massive transfusion and administration of selective coagulation products which even included recombinant factor 7 on one occasion.

## Discussion

The patient, a previously healthy young man, with fulminant Lyme myocarditis, refractory cardiogenic shock, and multiple organ failure (MOF) was successfully treated with different types of MCS in combination with comprehensive medical therapy immediately after admission to hospital. The possible fatal outcome was successfully avoided.

This is the first report of the Lyme carditis that was clinically presented as fulminant peri/myocarditis in young adult with favorable outcome. Usually, AHF due to Lyme carditis is manifested as non-fulminant myocarditis that generally has good prognosis if antimicrobial and other pharmacological therapy are appropriate and timely applied (10-22). In our case, myocarditis led to sudden onset of severe biventricular heart failure with rapid progression to cardiogenic shock and MOF. In addition, the patient had no previous or existing cutaneous signs of Borrelia infection and no history of a previous tick bite on admission to hospital. Nearly 60% of patients with symptomatic Lyme carditis do not have characteristic initial cutaneous manifestation of Borrelia infection, which is why early recognition of serious cardiac complications in Lyme disease is particularly important (12).

This is the first case report on the use of the MCS for Lyme carditis associated fulminant myocarditis with progressive biventricular AHF. We described a case of a successful treatment of the cardiogenic shock refractory to drug treatment that was followed by rapid development of MOF in young male patient with fulminant Lyme myocarditis. Treatment of the acute phase of the fulminant Lyme myocarditis based on the use of different modes of the MCS combined with appropriate antibiotic therapy for Borrelia infection resulted in complete recovery of the bilateral heart function. The MCS was integrated with aggressive pharmacological support and artificial ventilation early in the therapy during severe hemodynamic deterioration which occurred immediately after patient’s admission to hospital. Comprehensive cardiac assessment and continuous careful monitoring of cardiac function of the patients with fulminant Lyme myocarditis in respectable cardiac center where the MCS is available is prerequisite for successful bridging of the refractory cardiogenic shock in acute phase of the severe heart failure and for achieving sustained myocardial recovery. This was recently confirmed in many other cases of fulminant myocarditis of different etiologies (13-15).

Due to the hemodynamic instability, impaired oxygenation despite the use of artificial ventilation and unclear neurological condition due to pre-arrest state of the patient before the MCS implementation, we decided to use central VA ECMO as the first choice of circulatory assist device modality being a “bridge to recovery or bridge to decision”. This is in agreement with recent reports showing that short-term MCS, ECMO, has been predominantly used as an initial method of circulatory assist device vs long term, paracorporeal biventricular assist device (BiVAD) or LVAD, both in children and adult patients with fulminant myocarditis of other etiologies (16). The patient quickly restored hemodynamic stabilization and adequate oxygenation. In our case we used central VA ECMO for the first 10 days of treatment and then changed it into paracorporeal BiVAD for the next 14 days as there were no signs of satisfactory myocardial function recovery without evidence of significant damage of other vital organs. Paracorporeal BiVAD was used as the “bridge to recovery” or “bridge to transplantation”. Finally, only LVAD was used for two days during MCS weaning procedure. Successful bridging of acute phase of fulminant Lyme myocarditis was achieved with implementation of the MCS, which, upon restoration of bilateral ventricular function, was discontinued after the first 26 days of critical care. Further treatment, even after ventricular recovery, was directed according to standard evidence based AHF medications. It is well known that early implementation of MCS in the treatment of refractory cardiogenic shock due to fulminant myocarditis of different etiology contributes to the restoration of hemodynamic stabilization and satisfactory oxygenation and to faster recovery of both ventricular failure, too. Ventricular wall stress is decreased and geometry and cardiomyocyte function are improved with timely MCS support (17,18). Implementation of MCS increases survival in patients with fulminant myocarditis. This was confirmed in a series of case reports in patients with viral, peripartal, toxic, autoimmune, gigantocellular, necrotizing eosinophilic and idiopathic fulminant myocarditis with 68%-73% survival in adult patients after MCS discontinuation (19).

Early implementation of MCS is closely related to unsuccessful escalation of multiple vasoactive drug therapy and refractory cardiogenic shock, such as in our case. In addition, the period between the onset of symptoms of MOF and the implantation of MCS during the stage of imminent circulatory collapse positively correlates with the duration of the interval for LV recovery (20). In this case MOF developed before the MSC implementation, which was an additional risk factor of the worse outcome.

Upon confirmation of acute *Borrelia burgdorferi* infection, specific antimicrobial treatment with intravenous ceftriaxone was instituted from the fourth day of the ICU care and lasted for three weeks in total. Limitation of our case is the fact that we revealed diagnosis and started the appropriate antibiotic therapy of Lyme carditis on the fourth day of critical care. This highlights the importance of assessment of all patients with unexpected and uncommon cardiac symptoms for possible manifestation of Lyme carditis in high-incidence regions for Borreliosis and, conversely, evaluation of all patients with Lyme disease for cardiac signs and symptoms.

Cardiovascular symptoms in disseminated Borrelia infection are very rare and its prevalence is about 1% of all reported cases (21). Although, generally there is no gender difference in the incidence of Lyme disease, cardiac manifestations are three times more often in males than female (4,22).

Cardiac arrhythmias and conduction disturbances are the most common manifestations of symptomatic Lyme carditis (23). However, broad spectrum of less common cardiac complications such as endocarditis, pericardial effusion, myocardial infarction, sudden cardiac death, QT interval prolongation, repolarization disturbance, long-standing dilated cardiomyopathy have been described as a consequences of Lyme disease (24-26). Lyme carditis has been shown to have different manifestations, clinical presentations, treatments, and outcomes (Table 1).

**Table 1:** Manifestations of Lyme carditis: clinical presentations, treatment and outcome.

References	Manifestation of Lyme carditis	Treatment	Outcome
Koene R et al, 2012 (5)	Acute heart failure	Drug treatment	Recovery
Clinckeart et al, 2016 (27)	Perioperative biventricular heart failure resembling acute myocardial infarction	Drug treatment	Recovery
Muehlenbachs et al, 2016 (28)	None	None	5 sudden cardiac deaths
Yoon et al, 2015 (29)	Pancarditis	Drug treatment	Death
Rastogi et al, 2016 (30)	Fast AF/AU and transient AV block	Drug treatment	Recovery
Manek et al, 2014 (31)	Third degree AV block	Drug treatment and pacing	Recovery
Shenthar 2014 (32)	Third degree AV block	Drug treatment	Recovery
Center for Disease Control and Prevention; USA Department of Health and Human Service ; 2013 report (21)	None	None	3 sudden cardiac deaths
Patel et al, 2013 (33)	AV block, acute mitral regurgitation and acute coronary syndrome	Drug treatment	Recovery
Suresh et al, 2009 (34)	Third degree AV block	Drug treatment	Recovery
Tavora et al, 2008 (35)	Second-degree AV block	None	Death
Rostoff et al, 2008 (36)	Acute coronary syndrome	Drug treatment	Recovery
Greenberg et al, 1997 (37)	Fascicular tachycardia	Drug treatment	Recovery
Reimers et al, 1993 (38)	Cardiac arrest in the context of myositis	Drug treatment	Death
Artigao et al, 1991 (39)	Irreversible total AV block	Drug treatment and pacing	Recovery
Cary et al, 1990 (40)	Acute heart failure and third-degree AV block	None	Death
McAlister et al, 1989 (41)	Second/third degree AV block	Drug treatment and pacing	4 recoveries
Kirsch et al, 1988 (42)	Acute heart failure and ARDS	Drug treatment	Death
Marcus et al, 1985 (43)	Acute heart failure	Drug treatment	Death
Dernedde et al, 2002 (44)	Acute coronary syndrome	Drug treatment	Recovery

However, in some very rare cases, Lyme carditis can be life-threatening. Recently, Clinckaert at al (27) reported about a case of AHF presented with cardiogenic shock and repolarization abnormalities which mimicked acute coronary syndrome, rapidly followed by supra- and ventricular arrhythmias and severe heart block due to Lyme myocarditis manifesting at perioperative setting. The patient was successfully treated with multiple drug therapy. Also, in cases of sudden cardiac death in high-incidence Borreliosis areas fulminant Lyme myocarditis as well as severe cardiac blocks should be considered (27). Nearly 20% of all sudden deaths among young adults are considered a result of a myocarditis different etiology. Several cases of sudden cardiac death in adolescents and young adults were described as a consequence of undetected but extensive Lyme pancarditis confirmed at postmortem examination. Thus, Muehlenbachs et al (28) reported about five fatal Lyme pancarditis cases with subsequent sudden cardiac death in young adults, of whom four men, who all lived in high-incidence Lyme disease USA states. The authors also emphasized that the frequency of sudden cardiac death in systemic Borreliosis can be seriously under-recognized and underestimated in the above-mentioned areas. In a case report, Yoon et al (29) described a fatal case of Lyme fulminant carditis in a 17-year old patient who died unexpectedly after a 3-week viral like syndrome and on post-mortem examination diffuse pancarditis was revealed.

In conclusion, Lyme carditis can clinically present with fulminant myocarditis. In high-incidence regions for Borreliosis, physicians should be aware of the risk of Lyme myocarditis, especially in young adult males with unexpected cardiac symptomatology. If cardiogenic shock due to Lyme myocarditis does not respond rapidly to vasoactive medications, MCS should be considered in the early stage of the profound circulatory collapse. The MCS applied in the early stage of the pharmacological resistant cardiogenic shock contributes to rapid restoration of the hemodynamic stability, faster recovery of the bilateral cardiac function, as well as to significant higher odds for survival. Since MCS could be essential in the therapy of Lyme myocarditis associated with AHF, immediate transfer of the patients with this life-treating disease to a cardiac center with such possibility is mandatory.
